# The translational aspect of complementary and alternative medicine for cancer with particular emphasis on Kampo

**DOI:** 10.3389/fphar.2015.00150

**Published:** 2015-08-06

**Authors:** Marie Amitani, Haruka Amitani, Robert A. Sloan, Hajime Suzuki, Nanami Sameshima, Akihiro Asakawa, Yasuhito Nerome, Tetsuhiro Owaki, Akio Inui, Etsuo Hoshino

**Affiliations:** ^1^Education Center for Doctors in Remote Islands and Rural Areas, Kagoshima University Graduate School of Medical and Dental SciencesKagoshima, Japan; ^2^Department of Psychosomatic Internal Medicine, Kagoshima University Graduate School of Medical and Dental SciencesKagoshima, Japan; ^3^Division of Kampo Support, Cancer Institute HospitalTokyo, Japan

**Keywords:** complementary and alternative medicine (CAM), Kampo, Hozai, cancer, Hochu-ekki-to, Juzen-taiho-to

## Abstract

Complementary and alternative medicine (CAM) including Japanese Kampo is known to have anticancer potential. An increasing number of cancer survivors are using CAM for disease prevention, immune system enhancement, and symptom control. Although there have been abundant previous clinical reports regarding CAM, scientific investigations aimed at acquiring quantifiable results in clinical trials, as well as basic research regarding CAM, have only recently been undertaken. Recent studies suggest that CAM enhancement of immune function is related to cytokines. This review provides a translational aspect of CAM, particularly Hozai in Kampo from both scientific and clinical points of view for further development of CAM for cancer treatment.

## Introduction

Common side effects of cancer and cancer treatments that patients try to manage are related to the domains of mental and physical health-related quality of life (HRQoL). Although many patients with cancer are suffering, they may not be fully aware of less conventional and potentially alleviating resources (Carlson et al., 2004). To provide a better understanding of these broad resources, the National Center for Complementary and Alternative Medicine has categorized complementary and alternative medicine (CAM) to include health care systems, practices and products/medicines related to mind and body well-being (Monti et al., 2008).

Cancer patients have used CAM for the purposes of wellness, immune function, disease prevention and pain management. Notably 20–30% of cancer survivors reported using CAM for cancer prevention, treatment, symptom management, psychological distress, and other conditions (Sohl et al., 2014). Recent surveys have indicated that among cancer survivors and those without cancer, respectively 2/3 and 1/2 used CAM in their lifetime. However, when asked if they used CAM within the last year, about 2/5 from both groups reported use, but the highest use of CAM (∼3/4) has been reported from cancer patients during cancer therapy (Eng, 2010). These surveys revealed that cancer survivors are more likely to use CAM when compared with healthy people (Mao et al., 2011; Sohl et al., 2014). In another study, it was found that 1,666 long-term cancer survivors were: predominately female (62%), older (mean age = 69.5 years) and had breast, prostate, colorectal, ovarian, and endometrial cancers. Thus, when a cancer patient is treated, CAM is selected as one of the core treatments to support a patient’s HRQoL.

Deng et al. (2009), “the evidence-based clinical practice guidelines for health care providers” were issued by the Society for Integrative Oncology to help decide when to incorporate complementary health approaches into the care of cancer patients. Some studies note that when some conventional therapies and CAM approaches are used jointly, the control of cancer symptoms and treatment side effects might improve patient HRQoL (Deng and Cassileth, 2005; Deng et al., 2009). Other studies suggest that some complementary approaches, such as herbs, Kampo, acupuncture, natural food including ginger, massage therapy, yoga, and mindfulness-based stress reduction may help to control symptoms experienced by cancer patients and caused by cancer chemotherapy (Mehling et al., 2007; Ledesma and Kumano, 2009; Elkins et al., 2010; Cramer et al., 2012).

In Japan, CAM has been used predominantly in a clinical setting (Yamashita et al., 2002; Hottenbacher et al., 2013). The most commonly used therapeutic modalities in Japan are massage, vitamins, natural foods, dietary supplements, acupressure, and Kampo (Hori et al., 2008).

Although the majority of CAM users felt that CAM treatment was effective, almost two-thirds of cancer patients did not seek information or guidance from their physician (Hyodo et al., 2005). Additionally, clinical oncologists do not agree on the benefits of CAM as related to cancer treatment, because they consider CAM treatment options to lack sufficient evidence. Besides, they are concerned about the likelihood of drug interactions with an anticancer drug. Thus oncologists do not recommend CAM treatments to patients, and they do not recommend quitting CAM if the patient was already using it (Hyodo et al., 2003).

The number of clinical studies investigating the therapeutic benefits of CAM is increasing, therefore we will focus on clinical studies and basic research regarding CAM that might further the development of potential new therapies for cancer patients.

## The Clinical Use of Kampo for Cancer Patients in Japan

The theories and the methods of diagnosis in Japanese herbal medicine (Kampo) are distinctly different from Western medicine (Yu et al., 2006). Though Kampo originated from traditional Chinese medicine, it was adapted to Japanese culture (Yu et al., 2006). Kampo doctors in Japan have prescribed hundreds of Kampo formulas and the Ministry of Health, Labor and Welfare have approved nearly 150 formulas to date. There are numerous Kampo components used by Kampo doctors in Japan, and 2–15 of uniform parts are in every Kampo formula (Efferth et al., 2007). The majority of formulas are produced via a strict manufacturing process (Ohtake et al., 2004), and understanding the pharmacological mechanisms to analyze the active compounds in Kampo formulas with scientific methods is vital.

Since each Kampo formula consists of many types of compounds with different physicochemical properties, it’s hard to obtain an overall view of Kampo. However, progress in methods of scientific analysis has now made it possible to identify each Kampo formula by using the “fingerprint pattern” provided by three-dimensional high performance liquid chromatography (3D-HPLC) analysis (Ohtake et al., 2004). Kampo formulas are effective to alleviate the symptoms of cachexia, such as fatigue and anorexia, and they also reduce pain and improve the gastrointestinal side effects of anti-cancer drugs, including: diarrhea, nausea, and vomiting (Qi et al., 2010).

A majority of physicians (∼64%) surveyed reported prescribing Kampo medicines to help control cancer-related symptoms such as numbness/hypoesthesia, constipation, anorexia/weight loss, muscle cramps, and languor/fatigue. Kampo treatment was also found to extend significantly the survival of patients with uterine cervical cancer (Takegawa et al., 2008; Gao et al., 2012). The primary concern of physicians thus far has been in regards to dosage regulation (Iwase et al., 2012).

Western medicine uses a diagnosis approach based on the disease and symptomology while Kampo emphasizes patient-based diagnosis (Yu et al., 2006). In this way, “Kampo uses a treatment ‘formulation corresponding to Sho”’ where “Sho is the patient’s symptoms at a given moment” (Yu et al., 2006). When treating a patient, Japanese practitioners use a Sho diagnosis to tailor the Kampo formula (Yu et al., 2006). Hozai is selected according to ‘Sho,’ i.e., the patient’s condition from the Kampo point of view. Four diagnostic procedures of observation are necessary to determine the optimum formula: listening/smelling, questioning, and evaluation of pulse and abdominal patterns. When the patient has psychological symptoms such as insomnia, depression or irritation, together with autonomic nervous system symptoms such as gastrointestinal troubles and moist skin, Hochu-ekki-to is selected. If the patient does not have such symptoms and the skin is dry, Juzen-taiho-to or, especially when the patient complains of respiratory symptoms such as cough and shortness of breath, Ninjin-yoei-to is selected.

Hoshino et al. (2012) described ‘Hozai’ and ‘Sho’ as follows. Hozais are a group of formulas that invigorate patients who have lost mental and physical energy due to various causes including cancer. Kampo formulas containing *Ginseng radix* and *Astragali radix* are called ‘Jingizai’ (*Ginseng* and *Astragali* containing drug group) and are typical Kampo Hozais. Hochu-ekki-to, Juzen-taiho-to, and Ninjin-yoei-to are the three major Hozais and are the ones most frequently used for cancer patients. Cancer survivors suffer from several side effects of anti-cancer therapy, including effects of chemotherapy, radiation therapy, and hormone therapy. Numerous reports have shown the impact of Hozai in improving the HRQoL of cancer patients (Horie et al., 1994).

## Kampo and Cytokine in Cancer

The number of articles concerning the effects of Kampo formulas on cancer patients is on the increase (Satoh et al., 2002; Molassiotis et al., 2009; Okumi and Koyama, 2014; **Table 1**). Many of these studies have focused on the effects of medicinal herbal combinations on cytokine activity. These studies demonstrated that herbal mixture extracts have effects on at least one cytokine. The most commonly studied cytokines were interleukin (IL)-4, IL-6, IL-10, tumor necrosis factor (TNF)-alpha and interferon (IFN)-gamma (Burns et al., 2010). The therapeutic success of many formulas may be somewhat due to impacts on cytokines, which have revealed changes in their gene and protein expressions (Burns et al., 2010).

**Table 1:** Efficacy of herbal medicine.

Formula	Model	Reported outcome	Study	Reference
Hochu-ekki-to	Hepatoma	G0/G1 arrest	*In vitro*	Kao et al. (2001)
Hochu-ekki-to	Peripheral blood mononuclear cells (PBMC)	Stimulation of PBMC to produce G-CSF and TNF-alpha	*In vitro*	Kao et al. (2000)
Hochu-ekki-to	*Listeria monocytogenes*	Enhancement of IFN-γ production from infant CD4+ T cells	*In vivo*	Yamaoka et al. (2001)
Hochu-ekki-to	Ear swelling	Suppression of the increase in IL-4 by 2,4,6-trinitro-1-chlorobenzene	*In vivo*	Nakada et al. (2002)
Hochu-ekki-to	Cancer	Enhancement of NK activity	*In vivo*	Cho et al. (1991)
Hochu-ekki-to	Asthma	Reduction in Th2 responses (IL-4↓, -5↓)	*In vivo*	Ishimitsu et al. (2001)
Hochu-ekki-to	Endometrial carcinoma	Decrease in the expression of c-jun, TNF-alpha and estrogen receptors (ERs)-alpha and -beta	*In vivo*	Onogi et al. (2006)
Hochu-ekki-to	Ovalbumin – entrapped biodegradable microparticles	Enhancement of the mucosal IgA antibody response	*In vivo*	Matsumoto et al. (2010b)
Hochu-ekki-to	Breast cancer	Stimulated phosphorylation of c-Jun NH2-terminal kinase	*In vivo*	Volate et al. (2009)
Hochu-ekki-to	Colon cancer	Amelioration of cachexia	*In vivo*	Yae et al. (2012)
Hochu-ekki-to	Influenza	Increase in the production of GM-CSF	*In vivo*	Dan et al. (2013)
Hochu-ekki-to	Genitourinary cancer	Effective for anorexia	Clinical	Kuroda et al. (1985)
Hochu-ekki-to	*Mycosis fungoides*	Partial improvement in skin eruption	Clinical	Tokura et al. (1998)
Hochu-ekki-to	Cancer	Improvement in cancer-related fatigue	Clinical	Jeong et al. (2010)
Juzen-taiho-to	Human peripheral blood mononuclear cells	Increase in the production of GM-CSF	*In vitro*	Kubota et al. (1992)
Juzen-taiho-to	Hepatoma	Inhibition of hepatocarcinogenesis	*In vitro* and *in vivo*	Tatsuta et al. (1994), Ohnishi et al. (1998)
Juzen-taiho-to	Mononuclear cells and normal mice	Increase in the expression of IL-12 in the early stage, and IL-18	*In vitro* and *in vivo*	Fujiki et al. (2008)
Juzen-taiho-to	B16 melanoma	Increase in the production of IL-12	*In vivo*	Matsuda et al. (2011)
Juzen-taiho-to	Estradiol-related endometrial carcinoma	Suppression of estrogen-induced c-fos/jun-expression	*In vivo*	Niwa et al. (2001)
Juzen-taiho-to	Hepatoma	Inhibition of Kupffer cell-induced oxidative stress	Clinical	Tsuchiya et al. (2008)
Ninjin-yoei-to	Human peripheral blood mononuclear cells	Increase in the production of GM-CSF	*In vitro*	Okamura et al. (1991)
Ninjin-yoei-to	Healthy individuals	Enhancement of natural killer cell activity	Clinical	Kamei et al. (1998)
Ninjin-yoei-to	Lung carcinoma	Improvement in cough and anorexia Decrease in tumor marker (CEA and CA19-9) levels	Clinical	Kamei et al. (2000)
Ninjin-yoei-to	Pulmonary *Mycobacterium fortuitum* infection	Improvement in subjective symptoms Conversion to smear negative	Clinical	Nogami et al. (2006)
Rikkunshito	Cisplatin-induced anorexia	Improvement in cisplatin-induced anorexia via hypothalamic ghrelin	*In vivo*	Yakabi et al. (2010)
Rikkunshito	Cancer-cachexia	Improvement in anorexia–cachexia, prolongation of survival and reduction in anxiety-related behaviors	*In vitro* and *in vivo*	Fujitsuka et al. (2011)
Rikkunshito	Stomach cancer	Improvement in anorexia and body composition	*In vivo*	Terawaki et al. (2013)
Rikkunshito	Esophageal cancer	Improvement in anorexia, mood, QOL, and ADL	Clinical	Seike et al. (2011)
Rikkunshito	Cisplatin-induced anorexia	Improvement in anorexia	Clinical	Ohno et al. (2011)
Rikkunshito	After gastrectomy	Improvement in gastrointestinal symptoms after gastrectomy	Clinical	Takiguchi et al. (2013)

*G-CSF, granulocyte-colony stimulating factor; TNF-α, tumor necrosis factor alpha; IFN-γ, interferon-gamma; CD4, cluster of differentiation 4; IL-4, interleukin-4; NK, natural killer; Th2, T helper 2; GM-CSF, granulocyte macrophage colony-stimulating factor; IL-12, interleukin-12; IL-18, interleukin-18; CEA, carcinoembryonic antigen; CA19-9, carbohydrate antigen 19-9; PC-SPES, prostate cancer-SPES; PSA, prostate specific antigen; QOL, quality of life; ADL, activities of daily living.*

Numerous cytokines have been linked to symptoms of cancer patients. The equilibrium between proinflammatory and anti-inflammatory cytokines may play a significant function in the development of anorexia–cachexia syndrome. Proinflammatory cytokines—including IL-6, IL-1 beta, TNF-alpha and IFN-gamma—are possible mechanisms that may cause reduced caloric intake along with increased catabolic wasting, while anti-inflammatory cytokines, such as IL-10, IL-12, IL-4, and IL-15 have been found to have anti-cachectic properties (Amitani et al., 2013).

Proinflammatory cytokines can cause wasting, malnourishment, and eventually death by increasing the levels of the corticotropin-releasing factor (CRF; Ramos et al., 2004). Proinflammatory cytokines also provoke effects to mimic leptin signaling, which result in suppressed orexigenic ghrelin and neuropeptide Y. Therefore, anorexia and cachexia is induced, which is not counteracted by the usual compensatory response (Suzuki et al., 2013).

IL-6 plays an important role in anorexia–cachexia syndrome (Barton, 2005). IL-6 also regulates fat metabolism in muscle, which causes a rise in fatty acid oxidation and attenuates the lipogenic effects of insulin. Another proinflammatory cytokine, TNF-alpha, is reported to be increased in patients with cancer cachexia (Argiles et al., 2003). TNF-alpha binds to its receptor and induces activation of the nuclear factor (NF)-kappaB family of transcription factors (Von Haehling et al., 2002). Leukemia inhibitory factor induces anorexia by directly activating proopiomelanocortin neurons (Grossberg et al., 2010). Adrenocorticotropic hormone (ACTH) expresses leukemia inhibitory factor in the pituitary and prompts pro opiomelanocortin and ACTH release in response to inflammation (Chesnokova and Melmed, 2000).

Proinflammatory cytokines also induce cancer-related muscle wasting. It has been reported that acute and chronic central administration of IL-1 beta causes muscle atrophy (Braun et al., 2011). Thus, proinflammatory cytokines can induce central nervous system (CNS) inflammation, leading to muscle atrophy through stimulation of the hypothalamic–pituitary–adrenal (HPA) axis (Braun et al., 2011).

On the other hand, the anti-cachectic effect by anti-inflammatory cytokines has attracted a certain amount of attention. Researchers have shown that anti-inflammatory cytokines inhibit syndrome progression and prolong survival time by using the Myc/mTOR-driven animal model of anorexia–cachexia syndrome. This model has deregulated expression of cytokines, including IL-10 (Robert et al., 2012). The other anti-inflammatory cytokine, IL-15, increases glucose uptake in skeletal muscle and can be an anabolic factor for skeletal muscle (Busquets et al., 2006). IL-12 has been found to moderate body weight loss and other abnormalities associated with cachexia in mice with colon-26 carcinoma. Collectively, these findings begin to help shed light on the possibility of new therapeutic treatments for anorexia–cachexia syndrome (Amitani et al., 2013).

The number of reports on the interaction of herbal medicines with anticancer agents is also on the increase. Many Kampo products and active components have been reported to moderate an array of processes in cancer cell growth, invasion and metastasis by modulating a broad range of molecular targets, including: cyclooxygenase-2 (COX-2), NF-kappaB and NF erythroid 2-related factor 2-mediated antioxidant signaling pathways (Wang et al., 2010). The active components that have demonstrated chemopreventive effects may serve as potential principal agents for the design of new and less toxic agents for cancer chemoprevention (Wang et al., 2010).

*Ginseng* and *Astragalus*, which are contained in hozai, are reported its immunomodulatory function in preclinical and clinical studies (Keith and Mark, 2003). *Ginseng* stimulates NK cell and macrophage activities (Kim et al., 1990; Akagawa et al., 1996). *Ginseng* and *Astragalus* also influences cytokine secretion. *Ginseng* stimulates IL-2 and *Astragalus* produces IL-6 and TNF-alfa (Keith and Mark, 2003).

While each natural remedy has been revealed its effect, Kampo formula consists of multicomponent herbs. Since each herb has close interaction, Kampo formula shows its effect as total mechanism. The development of multicomponent herbal medicines, Kampo, may be valuable toward the potential management of complex diseases and new drug discoveries (Suzuki et al., 2012).

## The Translational Aspect of Each Hozai Formula

### Hochu-ekki-to

Hochu-ekki-to consists of: the herbs *Astragali radix, Atractylodis lanceae rhizoma, Ginseng radix, Angelicae radix, Bupleuri radix, Zizyphi fructus, Aurantii nobilis pericarpium, Glycyrrhizae radix, Cimicifugae rhizome*, and *Zingiberis rhizome*.

Hochu-ekki-to has been found to reduce *in vivo* tumor activity in rodent cancer models and treated syngeneic animals have also demonstrated repression in tumor growth. The splenic natural killer cells of rats treated with Hochu-ekki-to revealed a heightened cytotoxicity against tumors, along with the discovery of a potent inhibitory mechanism on tumor growth via the enhancement of natural killer cell activity (Cho et al., 1991). Another study showed that Hochu-ekki-to inhibited the spread of hepatoma cell lines by stimulating apoptosis and halting G0/G1 (Kao et al., 2001). In addition to the inhibitory effect of Hochu-ekki-to on tumor growth, immunological outcomes have been reported. One study regarding the immunological effects of Hochu-ekki-to showed that it stimulated peripheral blood mononuclear cells (PBMNCs) to produce granulocyte-colony stimulating factor (G-CSF) and TNF-alpha (Kao et al., 2000). Also, the temporal dose stimulation of murine normal colonic epithelial cell lines caused a significant increase in the levels of granulocyte colony-stimulating (Matsumoto et al., 2010a).

Hochu-ekki-to has also demonstrated inhibitory effects on endometrial carcinogenesis due to a decrease in the expression of c-jun, TNF-alpha, and estrogen receptors alpha and beta (Onogi et al., 2006). The oral administration of this herbal compound has also been shown to increase IFN-gamma secretion from lymphocytes, which increases L-selectin positive B lymphocytes, which is associated with boosting IgA immune response against intestinal antigens (Matsumoto et al., 2010b). These studies suggest that Hochu-ekki-to possesses chemotherapeutic effects.

Dose-dependent use of Hochu-ekki-to suppresses DNA synthesis and induces apoptosis that inhibit the spread of human breast cancer cell lines. Additionally, Hochu-ekki-to stimulated the phosphorylation of c-Jun NH2-terminal kinase (JNK), and pretreatment of breast cancer cells with the JNK inhibitor SP600125 eradicated apoptosis (Volate et al., 2009). Furthermore, combined treatment of Hochu-ekki-to and 5-FU improved the apoptotic effectiveness of 5-FU in breast cancer cell lines (Volate et al., 2009). These data suggested that Hochu-ekki-to has chemotherapeutic effects (Volate et al., 2009).

Hochu-ekki-to was shown to ameliorate cachexia induced by colon-26 adenocarcinoma cells in mice. Although the controlled treatment study did not demonstrate reduced tumor growth, it showed significant mitigation in the reduction of carcass weight, food and water intake. Also, the weight of the gastrocnemius and fat tissue around the testes and a decrease in serum triglyceride level was noted (Yae et al., 2012). The mechanisms by which Hochu-ekki-to mediated these effects were proposed to involve a reduction in the serum IL-6 and expression level in macrophages (Yae et al., 2012).

Hochu-ekki-to has also been shown to have a preventive effect against chemically induced carcinoma of the biliary tract (Tsuneoka et al., 2009) and to reduce cancer-related fatigue (Jeong et al., 2010).

While findings thus far are encouraging, more rigorous trials are needed to confirm the efficacy of Hochu-ekki-to for use in cancer patients.

### Juzen-taiho-to

Juzen-taiho-to consists of the herbs: *Astragali radix, Rehmanniae radix, Paeoniae radix, Angelicae radix, Poria radix, Glycyrrhizin radix, Ginseng radix, Cinnamomi cortex, Cnidii rhizoma*, and *Atractylodis lanceae rhizoma*.

Juzen-taiho-to was found to augment significantly the production of granulocyte-macrophage colony-stimulating factor (GM-CSF) but did not augment that of G-CSF by cultured human PBMNCs (Kubota et al., 1992). It was also used to treat animals with liver tumors, where it showed a significantly higher survival rate without any side effects compared to the untreated controls (Tatsuta et al., 1994; Ohnishi et al., 1998). It has been suggested that oral administration of Juzen-taiho-to inhibits liver metastasis of cancer through a mechanism that is mediated by activation of the host immune system, including activation of: macrophages, T-cells, B-cells, and natural killer cells (Ohnishi et al., 1998).

The cytokine IL-18 was the most notable up-regulated of the high number of cytokines that were assayed in the serum of patients with liver function disorders under long-term Juzen-taiho-to administration (Fujiki et al., 2008). It was also found that the oral administration of Juzen-taiho-to might stimulate the expression of IL-12 in the early stage, and IL-18 in the chronic phase, followed by natural killer-T cell stimulation. The mechanism of activation following immunological restoration could contribute to the anti-tumor effects (Fujiki et al., 2008).

The oral administration of Juzen-taiho-to in mice with B16 melanoma was found to inhibit tumor metastasis in the lungs. The suggested mechanism was the increase in the production of IL-12, which is responsible for the activation of both natural killer cells and natural killer-T cells in the lungs, resulting in inhibition of B16 melanoma cell metastasis in the lungs (Matsuda et al., 2011).

Despite the emerging literature, it is still not clear which of the compounds in Juzen-taiho-to handle the stimulation of individual cell types. Recently, Takaoka et al. (2014) reported a “biomarker-guided screening” method that identified intercellular adhesion molecule-1 by using a DNA array for macrophages via the purification of compounds responsible for stimulating macrophage activity of Juzen-taiho-to. The screening method resulted in the distillation of a glycolipid mixture containing beta-glucosylceramides, which stimulated intercellular adhesion molecule-1 expression in primary dendritic cells and primary CD14+ macrophages.

A preventive effect was demonstrated by using Juzen-taiho-to on endometrial carcinogenesis via the suppression of estrogen-induced c-fos/jun-expression that created an inhibitory effect on E2-related endometrial carcinogenesis in mice (Niwa et al., 2001). It was also demonstrated that four herbs of Juzen-taiho-to attenuated these effects significantly, and Shimotsu-to handled the inhibition of estrogen-related endometrial carcinogenesis (Lian et al., 2002).

Juzen-taiho-to was shown to reduce oxidative DNA damage, inflammatory cell infiltration and cytokine expression, and these, in turn, inhibited the development of liver tumors and the recurrence of hepatocellular carcinoma after surgery (Tsuchiya et al., 2008). Suppression of Kupffer cell function leading to a decrease in pro-inflammatory cytokines and oxidants in the liver was proposed to mediate the protective effect of Juzen-taiho-to against hepatocarcinogenesis.

Kampo formulas may be beneficial for cancer patients treated with chemotherapy or radiation therapy (Yamada, 1989). The combination of Mitomycin C and Juzen-taiho-to showed significantly greater anti-cancer activity and longer survival times than Mitomycin C alone (Komiyama et al., 1989; Yamada, 1989).

Juzen-taiho-to has been shown to reduce some of the side effects associated with anticancer drugs when combined with UFT and TS-1 treatments that contain a five-fluorouracil derivative. Combined treatment with Juzen-taiho-to mitigated weight loss (Sakamoto et al., 1991) and improved bone marrow suppression (Ogawa et al., 2012) in UFT colon cancer treatment in rats and TS-1 related cancer treatments, respectively.

Juzen-taiho-to creates radioprotective and anti-tumor effects by increasing the number and size of splenic CFU-S (Ohnishi et al., 1990). This anti-tumor effect has been markedly demonstrated when combined with surgical excision or with chemotherapeutic drugs. For example, a study that used combined Juzen-taiho-to therapy with IFN-alpha on experimental lung metastasis of murine renal cell carcinoma, resulted in a dose-dependent inhibition of lung metastasis after five consecutive treatment doses in mice (Muraishi et al., 2000).

### Ninjin-yoei-to

Ninjin-yoei-to consists of the herbs *Rehmanniae radix, Astragali radix, Angelicae radix, Ginseng radix, Polygalae radix, Glycyrrhizae radix, Paeoniae radix, Atractylodis rhizome, Poria cortex, Cinnamomi cortex, Aurantii nobilis pericarpium*, and *Schisandrae fructus.*

There have been a few investigations that have demonstrated the potential benefits of Ninjin-yoei-to. Researchers discovered that the compound increased natural killer cell activity after 2 days and lasted for a 1-week period in healthy people (Kamei et al., 1998). A case study reported decreased tumor CEA and CA19-9 markers in an older female with lung cancer after a 7-weeks treatment. And her cough was eliminated along with an improvement in appetite (Kamei et al., 2000). Cultured human PBMNCs in the presence of Ninjin-yoei-to *in vitro* demonstrated marked augmentation of GM-CSF production but not G-CSF (Okamura et al., 1991).

### Seisho-ekki-to

Seisho-ekki-to consists of the herbs: *Atractylodis lanceae rhizoma, Ginseng radix, Ophiopogonis tuber, Astragali radix, Aurantii nobilis pericarpium, Angelicae radix, Phellodendri cortex, Glycyrrhizae radix*, and *Schisandrae fructus*.

Seisho-ekki-to has been shown to have above average anti-oxidation activity. The effects of the combination of Seisho-ekki-to and *Scutellaria baicalensis* were evaluated in cancer-induced cachectic mice upon receiving chemotherapy with 5-FU. The combined application resulted in a significant decrease in tumor masses and losses of carcass and/or gastrocnemius muscles (Wang et al., 2012) and increases in both the Th1/Th2 ratio, NK cytotoxicity, and decreases in the expression of NF-kappaB and muscle RING finger protein-1 (MuRF-1) in the gastrocnemius muscle.

### Rikkunshi-to

Rikkunshi-to has been used to treat various gastrointestinal tract disorders and includes the herbs: A*tractylodis lanceae rhizoma, Ginseng radix, Pinelliae tuber, Poria cocos, Zizyphi fructus, Aurantii nobilis pericardium, Radix Glycyrrhizae, Zingiberis rhizome*, and *Atractylidis lanceae rhizoma*.

Tominaga et al. (2011) clarified the mechanism of Rikkunshi-to activity that underlies its clinical effect. It has been shown that it enhances gastric motility by causing an antagonistic effect on the 5-HT3 receptor. The hypothesis is that this may occur due to a singular transmission path that differs from that facilitated by the 5-HT3 receptor-antagonist (Tominaga et al., 2011). In a clinical study, Rikkunshi-to was demonstrated to significantly attenuate gastrointestinal symptoms after gastrectomy (Takiguchi et al., 2013). In use with animals and patients with cancer, it showed a reduction in anorexia, GI dysmotility, muscle wasting, anxiety and extended survival time (Fujitsuka et al., 2011). In a double-blind controlled trial in rats with cancer, it significantly ameliorated dysmotility-like dyspepsia and improved upper gastrointestinal symptoms (Hattori, 2010).

The combination of Rikkunshi-to with an anti-emetic drug has demonstrated some effectiveness against anorexia and vomiting induced by chemotherapy. It was recently indicated that it ameliorates cisplatin-induced anorexia by facilitating an increase in circulating ghrelin concentration (Yakabi et al., 2010). A recent RCT was conducted on advanced esophageal cancer patients who received combined chemotherapy with docetaxel/5-FU/CDDP and Rikkunshi-to. The combined treatment was effective in reducing the symptomatic occurrence of vomiting, nausea and anorexia. It also improved the quality of life, mood and activities of daily living (Seike et al., 2011). Another clinical study also showed that Rikkunshi-to reduced cisplatin-induced anorexia (Ohno et al., 2011).

In rats with tumors, a CRF receptor antagonist was shown to mitigate cancer anorexia–cachexia syndrome, and administration of the 5-HT2cR antagonist or Rikkunshi-to reduced hypothalamic CRF levels along with behaviors that are generally associated with anxiety that may lead to better quality of life (Fujitsuka et al., 2011). Another study found that the antrum and duodenum contraction rates in rats were improved after Rikkunshi-to treatment, owing to selective serotonin re-uptake inhibitor improvement via the enhancement of the circulating ghrelin and signaling (Terawaki et al., 2013).

These findings show that Rikkunshi-to may be useful for treating anorexia and may provide a new strategy for improvement of upper gastrointestinal dysfunction (Terawaki et al., 2014). Several studies have demonstrated that ghrelin or ghrelin receptor agonists were effective in the treatment of cancer cachexia (Fujitsuka et al., 2012). Rikkunshi-to has been shown to stimulate endogenous ghrelin secretion by blocking the 5-HT 2b/2c receptor pathway and enhancing ghrelin receptor activity (Fujitsuka et al., 2012). A study in rodents with tumors clarified that the active component hesperidin improved the effectiveness of ghrelin secretion while atractylodin improved receptor signaling and extended survival (Fujitsuka et al., 2011). Thus, Rikkunshi-to may provide a potential treatment method for anorexia, muscle wasting, and may prolong survival (Fujitsuka et al., 2012).

## Other Western Herbs Used as Cancer Treatments

In Western countries, herbs such as Iscador, Essiac, Pau D’Arco tea and cannabinoids are used commonly (Cheng et al., 2012). Also important is the herbal treatment Prostate Cancer-SPES (PC-SPES) that has taken attention for PC treatment. PC-SPES consists of eight natural substances; Reishi, Baikal skullcap, Rabdosia, Dyer’s woad, Saw palmetto, San-Qi Ginseng, Dendranthema morifolium, and Chinese licorice (Marks et al., 2002). For the past 20 years, men have used PC-SPES for prostate health. The combined name of this herb was developed from abbreviating the term PC and from the Latin word for hope, spes. PC-SPES first gained attention in a New England Journal of Medicine article that discussed its estrogenic effects on men and warned against unregulated use (Dipaola et al., 1998). In clinical case reports, PC-SPES decreased PSA levels, which increased after discontinuation of PC-SPES (Moyad et al., 1999; de la Taille et al., 2000; Oh et al., 2002; Olaku and White, 2011).

St. John’s wort is also an herb that has been commonly used by the public. A clinical study showed that the administration of St. John’s wort acted to decrease the plasma levels of SN-38 (the active metabolite of irinotecan) by 42% in cancer patients. In individuals without cancer, St. John’s wort has been shown to decrease the plasma levels of imatinib by 32% after 2 weeks of administration. Induction or inhibition of Cytochrome P450 or transporters was considered to be an essential mechanism (He et al., 2010).

Anticancer drugs that experience phase one/two metabolisms are found to be substrates of P-glycoprotein, breast cancer resistance protein and multidrug resistance-associated proteins. In combination with anticancer drugs, the mechanistic properties of these proteins are considered the key to herbal-anticancer drug interactions. However, more studies need to be done investigating harmful interactions (Yang et al., 2010).

Yet, when considering the herbal therapy used in the treatment of cancer patients, preclinical findings of clinical research must be considered. Both of risks and benefits of herbal therapy need to be discussed openly with patients (Eng, 2010). Thus, it is helpful to understand the effects and bioavailability of phytopharmaceuticals for developing new therapy approaches (Huang et al., 2008).

## Functional Foods

Maitake mushrooms (*Grifola frondosa*), have also demonstrated anticancer activity by increasing immune-competent cell activity that occurs from the combination of beta-1,6 glucan and beta-1,3 branched chains (Adachi et al., 1988; Hishida et al., 1988). An antitumor effect was found when the extract (MD-fraction) from maitake mushrooms was applied to rodents with tumors. The mechanism proposed was the activation of macrophages, T and natural killer cells that in turn enhanced the immune system (Inoue et al., 2002), and the effects have been found in other animal studies as well (Kodama et al., 2002). The use of whole maitake powder combined with MD-fraction was tested in a non-random case series on stage III-V cancer patients. More than half of liver cancer patients (∼58%), breast cancer patients (∼70%), and lung cancer patients (∼63%) experienced regression and improvements symptomology. Other combination approaches have also shown to be effective, chemotherapy plus maitake improved immune-competent cell activities ∼30% more than chemo alone (Kodama et al., 2002). The proposed mechanism of MD fraction to limit cancer progression comes primarily via the stimulation of natural killer cell activity (Kodama et al., 2003).

Green tea is the most widely consumed beverage after water. The major bioactive constituents are catechins, of which epigallocatechin-3-gallate, epicatechin, epicatechin-3-gallate, and epigallocatechin are the major components. The anticancer activities of green tea were highlighted recently (Zhang et al., 2014; Butt et al., 2015).

The catechin, epigallocatechin-3-gallate, has strong anti-oxidant and anti-inflammatory activity, and it affects several signal transduction pathways relevant to cancer development (Lambert et al., 2010). The anticancer effect of catechins is enhanced when they are combined with anticancer drugs (Suganuma et al., 2011; Yu et al., 2014). A large number of cohort studies and controlled randomized trials have suggested that green tea reduces the risk of cancer (Gao et al., 1994; Sasazuki et al., 2004). However, the opposite results—that there is no association between green tea consumption and the risk of cancer—have also been reported (Tsubono et al., 2001; Koizumi et al., 2003; Suzuki et al., 2004). The mechanisms by which green tea controls cancer insurgency have been found to occur in cell culture and animal studies. It is proposed that these mechanisms induce apoptosis, control cell growth arrest, alter the expression of cell-cycle regulatory proteins, activate killer caspases and suppress NF kappa-B activation (Butt et al., 2015). Based on the above reports, careful interpretation is needed when considering the preventive effects of green tea on cancer. Future intervention trials regarding functional foods including MD-fraction and green tea are warranted.

## The Mechanism and Management of Emotional Stress for Cancer

Mind-body therapies are growing in popularity due to the need of cancer patients to manage emotional stress (Elkins et al., 2010). Studies have evaluated therapies such as relaxation, biofeedback, meditation, hypnosis, yoga, art, music, and mind-body exercises such as Tai Chi and Qigong. These therapies have demonstrated various effects on improved mood, reduced pain, anxiety, insomnia and hot flashes, along with anticipatory and treatment-related nausea.

### Psychological Interventions and Immunity

A large number of studies have suggested a relationship between stress, coping factors and cancer (Monjan and Collector, 1977; Sklar and Anisman, 1979; Laudenslager et al., 1983; Chilvers and Peto, 1986; Chrousos, 1995). A recent study showed that chronic stress might cause social aversion via the glucocorticoid receptor in dopaminoceptive neurons (Barik et al., 2013). There are two major pathways activated by stressors: the HPA axis and the sympathetic-adrenal-medullary (SAM) axis (Chrousos, 1995; Glaser and Kiecolt-Glaser, 2005). CRF and ACTH found in high concentrations during stress, have a critical role in decreasing the production of many cytokines and inflammation mediators. Both mental stress and depression are considered to be associated with NK cell activities and decreased T-cell that affect processes of immune surveillance of tumors (Reiche et al., 2004).

The links between psychological states and cancer progression have been studied. The response of the HPA axis to stressors impairs the immune response by provoking the release of pituitary and adrenal hormones such as ACTH, cortisol, catecholamines, growth hormone and prolactin. The levels of these hormones are influenced by negative events or emotions (Glaser and Kiecolt-Glaser, 2005). The HPA axis and the SAM axis act directly or indirectly on cell surface receptors by inducing dysregulation of the production of cytokines such as IL-6, IFN-γ, IL-1, and TNF-alpha. Many studies suggest that psychological stress affects various aspects of the immune response at cellular, molecular and genetic levels via the CNS, hormones, cytokines, several neurotransmitters and neuropeptides. The neuroendocrine and immune system share several mediators and receptors, and they regulate the immune system. CRF has a major role in regulating psychoneuroimmunology. CRF affects the HPA axis and stimulates elevation in stress hormone levels, which in turn leads to deregulated immune function (Glaser and Kiecolt-Glaser, 2005). In turn, CRF activities in HPA are stimulated by proinflammatory cytokines (Shintani et al., 1993; Chen et al., 2010).

Serotonin also plays a key role in the progress of cancer anorexia (Rossi-Fanelli and Laviano, 2003). Increased levels of the serotonin precursor, plasma and brain tryptophan may be related to the increased serotonergic activity seen in the cancer anorexia–cachexia syndrome. A recent study revealed the role of hypothalamic 5-HT-CRF receptor pathway, which regulates ghrelin secretion in cancer anorexia–cachexia (Fujitsuka et al., 2011; Suzuki et al., 2013). Both 5-HT(1a) and 5-HT(2c) receptors influence anxiety-like behavior (Holmes et al., 2003; Heisler et al., 2007). Currently, the effect of elevated ghrelin is unclear. Some studies report that it may induce anxiety while others state that it may produce an anxiolytic-like response in animals that enables coping with stress (Chuang and Zigman, 2010).

### Cytokine-Mediated Depression and Anxiety

Proinflammatory cytokines may be related to the risk of depressive symptoms, shown in patients with cancer (Numakawa et al., 2014). The responses of cortisol to stress show crucial psychosomatic effects on the central nervous and immune systems as well as other tissues in the body. A cortisol treatment study also demonstrated a significant reduction in the production of IL-6 and generated an association with the impairment of memory recovery caused by emotional distress (Rohleder et al., 2009).

It has also been reported that central administration of IL-1 beta caused sickness and depressive-like behaviors with the increase of IL-6 in an animal model, whereas the administration of IL-6 resulted in depressive-like behavior (Sukoff Rizzo et al., 2012). These studies suggest that different cytokines are involved in both depression and sickness behaviors. Particularly elevated IL-6 may play a key role in the development of depression.

It has also been reported that a negative emotiogenic exposure can be related to specific immune reactions (Kalinichenko et al., 2014a). Furthermore, reduction of pro-inflammatory cytokine levels in active rats exposed to stress was less pronounced after intraperitoneal preinjection of melatonin. In passive animals, exogenous melatonin inverted the post-stress changes in the serum levels of the pro-inflammatory IL-2 cytokine and anti-inflammatory IL-4 and IL-10 cytokines (Kalinichenko et al., 2014b).

It was shown in a clinical study that emotional symptoms are related to the levels of several cytokines, such as IL-6, in several diseases, including rheumatoid arthritis and hemodialysis patients (Montinaro et al., 2010). Colorectal cancer patients had increased serum levels of IL-6, IL-1beta, TNF-alpha, and IL-8 but lower IL-10 concentrations. A correlation analysis of the Hospital Anxiety and Depression Scale score and cytokine levels revealed positive associations with the domain of anxiety/depression with IL-6, IL-1beta and TNF-alpha and a negative correlation with IL-10. These results suggested that circulating proinflammatory cytokines might be involved in the pathophysiology of anxiety and depression in cancer patients (Oliveira Miranda et al., 2014).

## Mind and Body Medicine

### Yoga

Yoga is a mind-body exercise that combines mind, body and breath, and users tend to be female with higher levels of education (Park et al., 2013). One study found that despite the interest in a yoga therapy program for cancer survivors, there were implementation barriers (Slocum-Gori et al., 2013).

Yoga therapy has demonstrated beneficial effects on improvement of components of health-related quality of life and symptom indices in survivors of breast cancer, survivors of non-small cell lung cancer and in patients undergoing radiotherapy (Mustian et al., 2013; Chandwani et al., 2014; Fouladbakhsh et al., 2014; Martin and Keats, 2014). Components of health-related quality of life were evaluated using the FACT-B survey and the breast cancer-specific well-being and Trial Outcome Index. Significant improvement was found in physical, social, emotional, and functional well-being but not social well-being (Levine and Balk, 2012). In a 12-weeks restorative yoga intervention, inflammation-related gene expression was reduced in breast cancer survivors (Bower et al., 2014). Other studies suggested the effectiveness of yoga for women with secondary arm lymphoedema from breast cancer treatment (Loudon et al., 2012, 2014). While there may be some apparent benefits in cancer patients that use yoga therapy, more research remains to be done, and the mechanisms by which yoga induces these effects need to be understood.

### Mindfulness-Based Stress Reduction

“Mindfulness” can be described as an engagement in a special form of meditation and a state of consciousness, depicted as a broadminded moment-to-moment awareness and a curious experiential openness and acceptance toward one’s own experiences (Bishop et al., 2004). The origin of mindfulness techniques, as used in psychosocial care, is derived from Siddhartha Gautama, the historical Buddha. Today the most commonly used mindfulness-based interventions are the mindfulness-based stress reduction (MBSR) and mindfulness-based cognitive therapy (Baer, 2003). The evidence that MBSR supports patients with cancer has been explored. Many outcomes support the effect of MBSR in improvement of mood, stress, anxiety, fear of recurrence, adjustment, HRQoL, and locus of control (Lengacher et al., 2012, 2014). The mechanism that has been suggested to underlie these effects is that MBSR reduces cortisol levels and the levels of cytokines such as IL-6 (Carlson et al., 2007; Witek-Janusek et al., 2008; Lengacher et al., 2012). A study demonstrated that MBSR participants versus non-participants had re-established levels of immunoregulatory factors, such as PBMNC natural killer cell activity and cytokine regulation, and the study additionally demonstrated a reduction in cortisol levels, improved HRQoL, and increased coping effectiveness (Witek-Janusek et al., 2008).

## Conclusion

Increased evidence has shown that CAM, especially Kampo and mind-body therapies, may serve as beneficial adjuncts to cancer treatment. Health professionals need to be aware of such evidence and to use these interventions as complementary treatments in an integrated way. Health professionals are also required to support patient evidence-based decision-making since patients want to try everything they can alleviate the burden of cancer. In this vein, further studies, particularly double-blind studies, are needed to verify the efficacy of CAM and to obtain high-quality evidence to support the use of CAM.

## Conflict of Interest Statement

The authors declare that the research was conducted in the absence of any commercial or financial relationships that could be construed as a potential conflict of interest.
